# Reply to Gillan et al.: What should be the null hypothesis in computational psychiatry?

**DOI:** 10.1073/pnas.2628596123

**Published:** 2026-10-16

**Authors:** Noam Sarna, Reuven Dar, Matan Mazor

**Affiliations:** ^a^ School of Psychological Sciences, Tel Aviv University, Tel Aviv 6997801, Israel; ^b^ All Souls College, University of Oxford, OX1 4AL Oxford, United Kingdom; ^c^ Department of Experimental Psychology, University of Oxford, OX1 3EL Oxford, United Kingdom

We thank Gillan et al. (1) for this exchange. To clarify, response biases and statistical artifacts are certainly not the only possible interpretation of existing data linking metacognitive biases and self-reported psychopathology. Substantive associations remain a viable alternative, and one that should be tested in future studies. Our main point is rather that, going forward, positive claims of such associations should rest on evidence that goes beyond what is expected from these nuisance factors alone.

In the case of response biases, we believe that current evidence does not meet this bar. The assertion of low inattention rates in the PIP (2) and Neureka (3) datasets rests on low inattention *detection* rates (PIP, 8.3%; Neureka, <1%), which are indeed well below the 24% we detect (4) and the 22% Zorowitz et al. (5) find in a clinical sample. These lower rates are likely affected by the detection method: PIP used instructed-response items (“if you are paying attention, select x”), a poor measure of inattention (6, 7), and Neureka a single infrequency item (in our study (4), a well-fitting model showed that a single infrequency item detects only 39% of inattentive participants, leaving 61% undetected; SI Appendix, figure A15). Because these confounds are far larger than the effect of interest [roughly by a factor of 10; (4, reply to Gillan, SI Appendix, section 2)], and since participants flagged as inattentive in the PIP study display exactly the profile attributed to compulsive individuals (elevated CIT, OCI-R, and confidence; Fig. 1*A*), the CIT–confidence association in these datasets remains consistent with the effect being entirely driven by residual response biases (8). This is not the only possible interpretation of the data, but it remains a viable one.

**Fig. 1.**
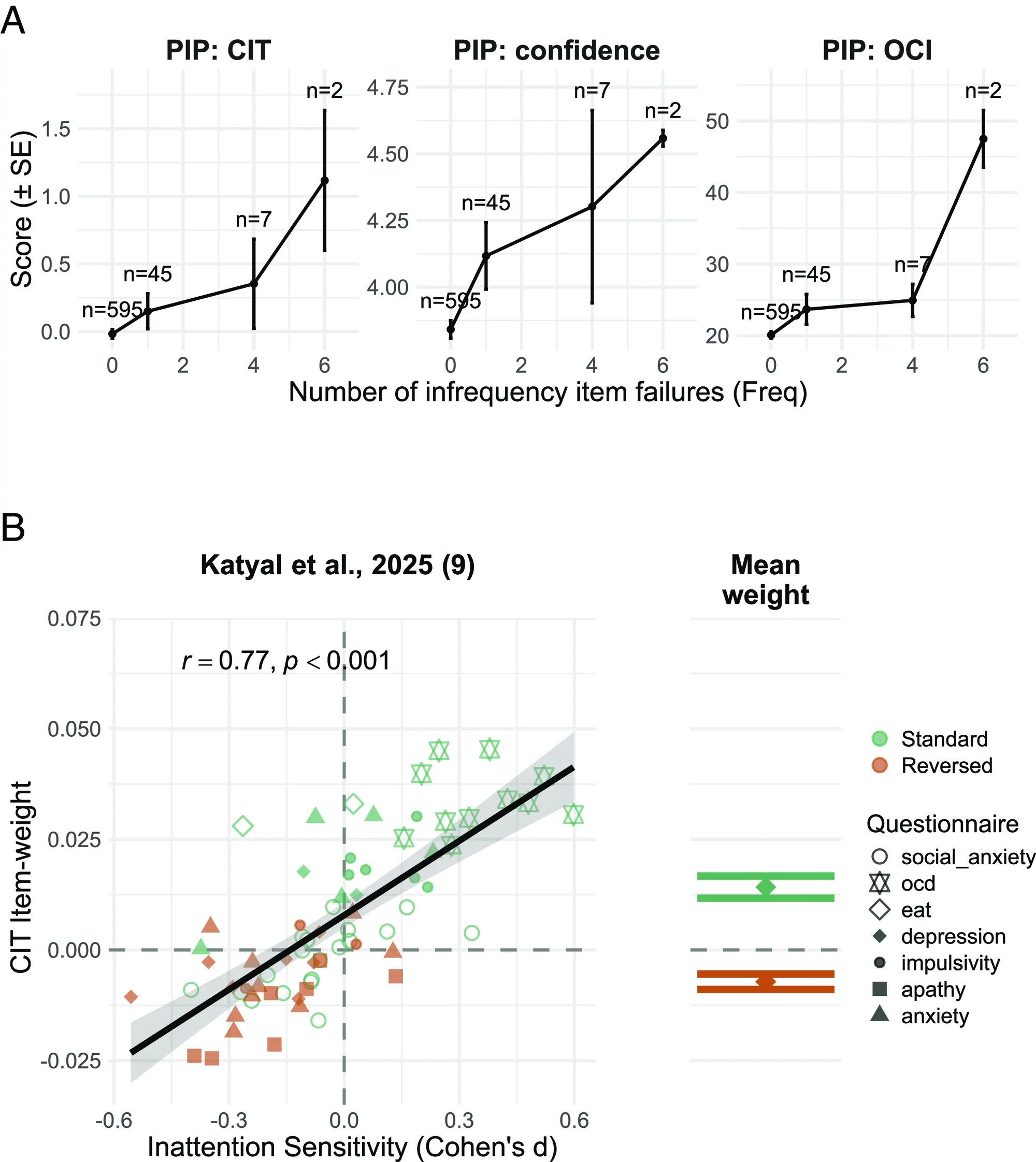
(*A*) Inattentive participants in PIP (2) show elevated CIT, confidence, and OCI-R scores. Adapted from Sarna et al. (4, SI Appendix, figure A17). (*B*) The effect of reversed items and inattention on the CIT factor in Katyal et al. (9). Each point is a questionnaire item (shape = questionnaire; color = standard vs. reversed). *x*-axis: item sensitivity to inattention (Cohen’s *d*, inattentive vs. attentive); *y*-axis: CIT item-weight. Item weights are assigned to reversed items after reversal. Diamonds (*Right*) show mean ± SEM weight for standard (green) and reversed (orange) items. Reversed items are used as safeguard against acquiescence. However, the CIT systematically assigns reversed items negative weights, effectively turning this safeguard into an additional source of bias. Adapted from Sarna et al. (4, figure 4), which is licensed under CC BY-NC-ND 4.0.

The same holds for covariate control. The emergence of a significant confidence–OCD association when controlling for a correlated covariate (depression) is compatible with a spurious correlation produced by a collider bias. Beyond a simulation, we showed in our own data that this mechanism produces a correlation between measures that are independent by construction: acquiescence and SDS, a questionnaire with 50% reversed items (4, reply to Gillan, SI Appendix, section 1). Similar to the case of response biases, whenever the observed pattern is compatible with a likely alternative explanation, we contend that this should serve as the null hypothesis to reject.

Finally, regarding the differences between CIT and OCI-R, we note that correlations with confidence can be driven by acquiescence, independent of inattention. While the OCI-R is affected by acquiescence only due to the absence of reverse-coded items, CIT is doubly susceptible: it includes reverse-coded items but systematically undoes the semantic reversal, assigning the already reversed scores a negative weight (Fig. 1*B*). This again makes differences in correlations with confidence difficult to attribute to psychopathology alone.

Gillan et al. (1) point to important next steps to address these open questions, and we agree. Specifically, new designs and measures that are less vulnerable to these confounds will more unequivocally tease apart substantive associations from the effects of response biases and statistical artifacts.

## Data Availability

Analysis code and data have been deposited in pnas-response (https://github.com/self-model/pnas-response/) (10). Previously published data were used for this work (2, 9).
